# Clinical-epidemiological features of HIV-infected patients diagnosed at age of 50 years or older

**DOI:** 10.7448/IAS.15.6.18108

**Published:** 2012-11-11

**Authors:** P Patterson, S Cahn, O Sued, V Fink, M Figueroa, C Cesar, M Rojo, G Ben, M Vázquez, P Cahn

**Affiliations:** 1 Fundación Huésped, Clinical Research, Buenos Aires, Argentina; 2 Fundación Huésped, Epidemiology and Prevention, Buenos Aires, Argentina; 3 Hospital Fernández, Infectious Diseases Unit, Buenos Aires, Argentina

## Abstract

HIV/AIDS prevention and care efforts are directed to individuals of reproductive age (15–49 yrs). With the extension of sexual life of older people, they became a growing population at risk of HIV infection, usually not included in prevention strategies. In order to evaluate clinical profile of HIV/AIDS pts diagnosed at 50 yrs or older assisted in an HIV outpatient center in Buenos Aires, we retrospectively assessed clinical records of pts initiating care between Jan 1986 and Dec 2011. Age, CD4 cells and viral load (pVL) at HIV diagnosis and most recent value, opportunistic infections (OIs), co-morbidities and antiretroviral therapy (ARV) were recorded. Of 10,998 pts assisted in the 26-yr period, 495 (4.5%) were≥50 yrs old at HIV diagnosis; median annual diagnoses: 18.5 (IQR 3.3–30.3) without significant changes in the last 20 yrs. Demographics: median age 54.7 yrs (IQR 51.8–59.2, rank 50–80), 76.6% male. Risk behavior: HTX 61.4%, MSM 34.1%, others 4.4%. 55.4% of HIV diagnoses occurred during hospitalization or simultaneously with acute OIs. One third (n=176) had AIDS at diagnosis, 24% had history of STDs. HCV co-infection 5.7%, past HBV infection 28.1% and chronic HBV infection 5.1%. Median CD4 cells at HIV diagnosis: 223.5 (13.7%) (IQR 98.8–420.3), initial pVL 60,000 cp/mL (IQR 9,995.5–208,391). 69.3% of pts started ARV therapy during follow-up (FU), and the median time between diagnosis and treatment initiation was 3.4 mo (IQR 0.7–14); 56.9% of them started a non-nucleoside-based regimen (ZDV/3TC/EFV), 28.3% a PI-based regimen (ZDV/3TC/IDV) and 14.6% a nucleoside-based regimen (ZDV/ddI pre-HAART era). After a year (±6 mo), 63.8% pts achieved undetectable pVL and gained 136 CD4 cells from BSL (IQR 83–204). After 40.6 mo of FU (IQR 6.7-89.8), 66.3% are alive, 7.1% died (68.6% of HIV-related diseases) and 26.7% are lost to FU. Co-morbidities were present in 125 (25.3%), mainly hypertension, increased lipids, CVD and DBT. Among treated pts, 70.6% achieved pVL<50 cp/mL, with a median increase of CD4 cells up to 410 (22%) (IQR 281.5–563.9) from BSL. 51% (176) changed ARV therapy due to toxicity/AE: 54.5%, ARV failure: 29.5% and simplification: 14.8%. Stable HIV epidemic in older people reinforce the need of specific prevention approaches, while growing age of HIV individuals in care highlights to consider risks associated to older age. Late presentation to care needs to be specifically addressed. Response to treatment is remarkable high in this population.

